# The three-dimensional coarse-graining formulation of interacting elastohydrodynamic filaments and multi-body microhydrodynamics

**DOI:** 10.1098/rsif.2023.0021

**Published:** 2023-05-31

**Authors:** Paul Fuchter, Hermes Bloomfield-Gadêlha

**Affiliations:** Department of Engineering Mathematics and Bristol Robotics Laboratory, University of Bristol, Bristol, UK

**Keywords:** elastohydrodynamics of filaments, microhydrodynamics, fluid–structure interaction, elastic filaments in three dimensions, cilia and flagella, microorganisms and microswimmers

## Abstract

Elastic filaments are vital to biological, physical and engineering systems, from cilia driving fluid in the lungs to artificial swimmers and micro-robotics. Simulating slender structures requires intricate balance of elastic, body, active and hydrodynamic moments, all in three dimensions. Here, we present a generalized three-dimensional (3D) coarse-graining formulation that is efficient, simple-to-implement, readily extendable and usable for a wide array of applications. Our method allows for simulation of collections of 3D elastic filaments, capable of full flexural and torsional deformations, coupled non-locally via hydrodynamic interactions, and including multi-body microhydrodynamics of structures with arbitrary geometry. The method exploits the exponential mapping of quaternions for tracking 3D rotations of each interacting element in the system, allowing for computation times up to 150 times faster than a direct quaternion implementation. Spheres are used as a ‘building block’ of both filaments and solid microstructures for straightforward and intuitive construction of arbitrary three-dimensional geometries present in the environment. We highlight the strengths of the method in a series of non-trivial applications including bi-flagellated swimming, sperm–egg scattering and particle transport by cilia arrays. Applications to lab-on-a-chip devices, multi-filaments, mono-to-multi flagellated microorganisms, Brownian polymers, and micro-robotics are straightforward. A Matlab code is provided for further customization and generalizations.

## 1. Introduction

**Figure 1.**
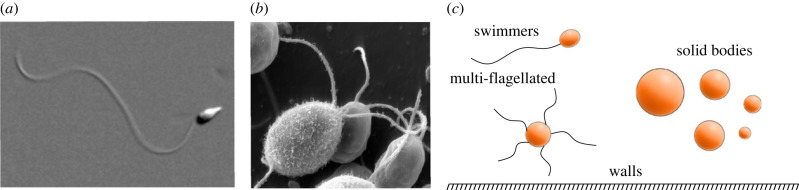
(*a*) Uni-flagellated sea urchin sperm [1]. (*b*) Multi-flagellated green algae *Chlamydomonas*. From https://www.dartmouth.edu/emlab/. (*c*) Representation of interactions that the method is capable of simulating: multiple filaments, filaments connected to arbitrary solid bodies, multi-filament–body structures and micro structures with arbitrary shapes, free or fixed.

Elastic passive and active filaments are the building blocks of numerous biological, physical, engineering and robotic systems. These include, but are not limited to, polymer and fibre dynamics, cilia driving fluid in the lungs, microtubules regulating deformations in cancerous cells, flagella propelling spermatozoa and algae (figure 1*a*,*b*), and robotic micro-swimmers in microfluidic devices, among many others [2–6]. Simulating these structures fully in three dimensions (3D) is challenging due to the convoluted balance of several interacting components. Moments arise from elasticity, internal activity, contact and the inertialess fluid that the filaments are embedded in. This complexity is augmented by the ‘non-local’ presence of other structures embedded in the fluid, such as walls and solid bodies. Numerous computational architectures have been developed to date attempting to simulate these systems [7–12], a testimony of the growing importance of filament fluid–structure interactions to the scientific community at large, and we direct the reader to the recent review on the topic by du-Roure *et al*. [2] and references therein.

A hierarchy of fluid and elastic theories exist from continuous to discrete, with varying levels of complexity depending upon the precision required. Methods for resolving inertialess hydrodynamics include local theories, such as resistive force theory (RFT) [13], and more complex non-local theories, such as slender-body theories (SBT), regularized Stokeslets [14], the Rotne–Prager–Yamakawa (RPY) approximation [15] and boundary-element methods (BEM) [16]. Similarly, elastic deformation of filaments may be described using Cosserat rod theory, which accommodates bending, twist, shear and extensibility in 3D [17], the Kirchoff rod approximation [18], where filaments can only bend and twist, or even constraining inextensible filaments to planar deformations (with zero twist) [19]. Immersed boundary methods exploit direct numerical solution of Navier–Stokes equations with a Lagrangian mesh for the filament [20], while in bead models the filament is discretized into segments, and the elastic coupling is described via discrete elastic bonds [21,22].

Classical formulations of solving filament elastohydrodynamics revolves around the continuum limit of moment balance [23,24]. They are critical for analytical progress and direct model interpretability from the resulting partial differential equations (PDEs) [19]. However, the PDEs have a high order, are highly nonlinear and are coupled with boundary value problems (BVP), which require numerous boundary conditions. Unsurprisingly, these equations are challenging to solve even in lower dimensions [24,25]. They are also numerically stiff and thus computationally expensive/time-consuming, often requiring penalization strategies to regularize numerical errors [23]. They also require specific numerical architecture that is not easily transferable to different applications, especially in 3D, nor easily generalizable to systems involving non-trivial geometry and/or possessing many, and distinct, interacting units, such as elastic filaments interacting with solid structures (figure 1*c*). As we shall see below, the formulation derived here will attempt to resolve these challenges.

Recent developments in coarse-graining (CG) formulations [12] offer intuitive model interpretability, straightforward implementation and numerical advantages over previous methods. By asymptotically integrating the momentum density over ‘coarse’ segments along the filament, the CG method arises from first principles, and removes the need to solve for unknown contact forces, Lagrange multipliers and associated boundary conditions to enforce filament inextensibility [12,26]. The elastohydrodynamic PDE is recast into a simpler set of ordinary differential equations (ODEs) with trivial and intuitive matricial form, only depending on the filament parametrization, as we explore further in this paper. Moreau *et al*. [12] highlighted the high efficiency of the formalism in two dimensions (2D), and showed excellent agreement with classical formulations, even for relatively large CG segments. Following this, the CG method has gained momentum, and was adopted and generalized in several ways: (i) non-local hydrodynamics have been accounted for passive and active filaments in 2D, including wall interactions [27–29], (ii) 3D filament deformations were accounted for in [30,31], though limited to a local hydrodynamic resistive force theory (RFT), as in [12], (iii) it has been applied to study sperm flagellum propulsion in 2D [32], (iv) expanded into a hybrid immersed boundary method [33], as well as a stochastic method [34], and (v) used for analytical and numerical study of the well-posedness of Newtonian and viscoelastic elastohydrodynamic propulsion [35,36].

Despite the above momentum in the literature, there is currently no CG formulation that allows simulation of collections of interacting elastohydrodynamic filaments with arbitrary solid structures in 3D. Here, we capitalize on the strengths of CG formulation and develop a fast simulation tool to resolve multiple interacting elastohydrodynamic filaments and solid body microhydrodynamics in 3D. Our generalized CG formulation is efficient, intuitive and easy to implement and customize. Our method allows for simulation of 3D elastic filaments with both flexural and torsional deformations, coupled hydrodynamically to free or fixed solid bodies and microstructures present in the environment (figure 1*c*). We use spheres as the hydrodynamic ‘building block’ of filaments and solid structures for straightforward application of the method to novel and arbitrary 3D geometries, though we note that any hydrodynamic theory may be employed instead. The method is validated against previous experiments and simulations in the literature.

We show that despite the substantial advantage of employing the CG formulation, runtimes can still be hindered by the choice of coordinate-system parametrization. We exploit the benefits of the exponential mapping of quaternions [37,38] for high-precision tracking of basis-rotations in three dimensions. This allows fast and efficient computation, up to 150 times faster than a direct quaternion parametrization. The exponential map also avoids undesired repeated change of basis used to circumvent coordinate-singularities introduced by Euler angles [18,30,31], critical while tracking multiple filaments simultaneously. Finally, we highlight the strengths of the new method in a series of non-trivial applications and report novel results. These include the beating of multi-flagellated swimmers, sperm–egg elastohydrodynamic scattering and particle transport by cilia arrays.

## 2. Methods

We consider collections of 3D inextensible and unshearable elastic filaments undergoing deformations of bend and twist (Kirchoff rods) that are embedded in a viscous inertialess fluid, interacting hydrodynamically with each other and with solid bodies and microstructures with arbitrary shapes, free or fixed, present in the micro-environment. We begin by outlining the CG formulation solving the dynamics of a single 3D elastohydrodynamic filament connected to a solid body, before generalizing to an arbitrary number of filaments and solid bodies.

### 2.1. The coarse-graining formalism in three dimensions

**Figure 2.**
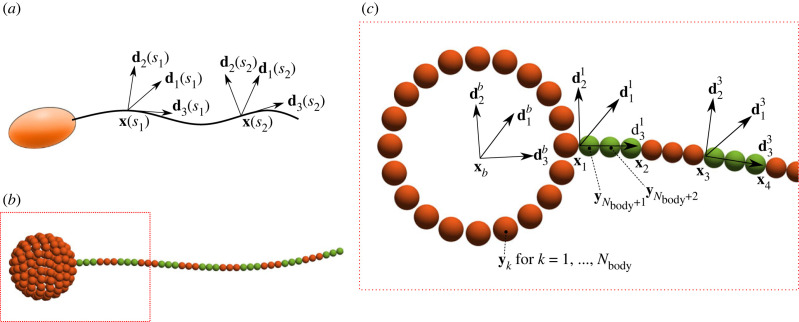
(*a*) An elastohydrodynamic structure comprising solid body and filament. In this example, the solid body represents the head of a swimmer and the filament represents a flagellum. The filament is described by centreline **x**(*s*) with director basis [**d**_1_(*s*), **d**_2_(*s*), **d**_3_(*s*)] shown at two arbitrary points *s* = *s*_1_ and *s* = *s*_2_. (*b*) The structure is made up of spheres. Body constructed using *N*_body_ = 133 spheres and filament using *N* = 13 rigid segments with *n* = 3 spheres per segment. (*c*) Body position **x**_*b*_ and body director basis $\left[d_{1}^{b},d_{2}^{b},d_{3}^{b}\right]$ labelled. Segment positions **x**_*i*_ labelled as well as the director basis for segments 1 and 3. Sphere positions are given by **y**_*k*_, with *k* ≤ *N*_body_ labelling body spheres and *k* > *N*_body_ labelling filament spheres.

We consider first a single Kirchoff filament attached to an arbitrary solid body (figure 2*a*). Let **x**(*s*) be the position of the centreline of the filament, where *s* is an arclength between *s* = 0 and *s* = *L*, where *L* is the length of the filament. To describe deformations of the filament we will use the orthonormal local director basis [**d**_1_(*s*), **d**_2_(*s*), **d**_3_(*s*)], defined at every point along the centreline. As such, **d**_3_(*s*) is tangent to the curve **x**(*s*) and given by **d**_3_(*s*) = ∂**x**/∂*s*, and **d**_1_(*s*) and **d**_2_(*s*) point normal to the curve **x**(*s*). Derivatives of the director basis with respect to arclength are related to the twist vector ***κ***(*s*) by

2.1 $$\frac{\partial d_{i}}{\partial s}=\kappa \times d_{i},$$

where ***κ*** = *κ*_1_**d**_1_ + *κ*_2_**d**_2_ + *κ*_3_**d**_3_. The constituents of the twist vector *κ*_1_, *κ*_2_ and *κ*_3_ are known as the filament curvatures [18]. The third curvature in the **d**_3_ direction can also be referred to as the twist density. We direct the reader to Goriely [39] for a thorough discussion on the interplay between the three components of the twist vector and the differences between *κ*_3_, torsion and twist.

The balance of forces and torques along the filament at low Reynolds number reads [40]

2.2 $$n_{s}+f=0$$

and

2.3 $$m_{s}+x_{s}\times n+\tau =0,$$

where **n**_*s*_ and **m**_*s*_ are the contact force and contact moment, **f**(*s*) and ***τ***(*s*) are the force and torque densities exerted on the filament by the fluid, and subscript *s* denotes differentiation with respect to *s*. The elastic moment is known constitutively for a Kirchoff rod, whereas the contact force remains unknown. The CG formalism is derived from first principles by exploiting the integral form of the momentum balance above. This circumvents the need for solving a high-order PDE with a second-order BVP using Lagrange multipliers [12]. For this, we discretize the body into *N*_body_ spheres and the filament into *N* rigid segments each made of *n* spheres, as shown in figure 2*b* for *N*_body_ = 133, *N* = 13 and *n* = 3. Positions of spheres in the body are defined relative to the frame of reference that moves with the entire structure, described by the local basis $\left[d_{1}^{b},d_{2}^{b},d_{3}^{b}\right]$ and located at **x**_*b*_ (figure 2*c*). Sphere positions are given by **y**_*k*_ for *k* = 1, 2, …, *M*, where *M* = *N*_body_ + *Nn* is the total number of spheres in the system. Body spheres are labelled by *k* ≤ *N*_body_ and filament spheres by *k* > *N*_body_. Segment endpoints are located at **x**_*i*_ for *i* = 1, 2, …, *N*, and **x**_1_ is defined relative to **x**_*b*_ in the local basis $\left[d_{1}^{b},d_{2}^{b},d_{3}^{b}\right]$. Each segment is oriented in space according to its local director basis $\left[d_{1}^{i},d_{2}^{i},d_{3}^{i}\right]$, for *i* = 1, …, *N*. As such, subsequent segment positions are given by

2.4 $$x_{i}=x_{1}+\Delta s\sum_{\;j=1}^{i-1}d_{3}^{j},$$

for *i* = 2, 3, ..., *N*, where Δ*s* = *L*/*N*, ensuring the filament remains exactly inextensible.

Invoking force-free and torque-free conditions, the total momentum balance of the elastohydrodynamic filament–body system in equations (2.2) and (2.3) reads

2.5 $$\sum_{i=1}^{N_{\mathrm{body}}+Nn}F_{i}=0$$

and

2.6 $$\sum_{i=1}^{N_{\mathrm{body}}+Nn}((y_{i}-x_{b})\times F_{i}+T_{i})=0,$$

where **F**_*i*_ and **T**_*i*_ are the total force and torque, respectively, exerted on sphere *i* by the fluid. Integrating equation (2.3) from *s* = *s*_*j*_ to *s* = *L* yields

2.7 $$\sum_{i=N_{\mathrm{body}}+(j-1)n+1}^{N_{\mathrm{body}}+Nn}((y_{i}-x_{j})\times F_{i}+T_{i})=-m_{j},$$

for *j* = 2, …, *N*, where **m**_*j*_ is the bending moment at *s*_*j*_ defined constitutively by

2.8 $$m_{j}=E_{b}(\kappa_{1}^{j}-\kappa_{1}^{0}(s_{j}))d_{1}^{j}+E_{b}(\kappa_{2}^{j}-\kappa_{2}^{0}(s_{j}))d_{2}^{j}+E_{t}(\kappa_{3}^{j}-\kappa_{3}^{0}(s_{j}))d_{3}^{j},$$

where *E*_*b*_ is the bending stiffness, *E*_*t*_ is the torsional stiffness, $\kappa_{\alpha }^{j}$ are the curvatures at *s*_*j*_ for *α* = 1, 2, 3, and $\kappa_{\alpha }^{0}(s_{j})$ is the preferred curvature at *s*_*j*_. If $\kappa_{\alpha }^{0}(s_{j})=0$ for *α* = 1, 2, 3, the filament will relax to a straight configuration. By making $\kappa_{\alpha }^{0}(s_{j})$ non-zero, the filament will relax to non-straight configurations, and by making $\kappa_{\alpha }^{0}(s_{j})$ a travelling wave, the filament can be made to swim. The CG governing equations (2.5)–(2.7) can be written in a simpler and compact matrix form, given by 2.9
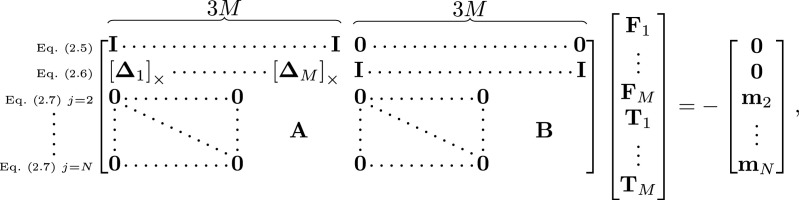
where we have used the matrix form of the cross product, denoted by ${\left[a\right]}_{\times }$ (see appendix A), and defined **Δ**_*i*_ = **y**_*i*_ − **x**_*b*_. The blocks of zeros multiply forces and torques experienced by spheres in the body, as they do not contribute to the bending moment relations on the filament. The matrices **A** and **B** multiply forces and torques experienced by the spheres in the filament, and are size 3(*N* − 1) × 3*Nn*. The matrix **A** comprises (*N* − 1)*Nn* sub-matrices given by $A_{i,j}={\left[y_{N_{\mathrm{body}}+j}-x_{i+1}\right]}_{\times },\text{ if }j>in$, and zero otherwise. The matrix **B** has the same structure as **A** with sub-matrices given by *B*_*i*,*j*_ = **I**, if *j* > *in*, and zero otherwise.

The CG matrix formulation in equation (2.9) encodes the force and torque balance for a single filament attached to an arbitrary solid body (figure 2*b*). By setting *N*_body_ = 0 or *N* = 0, we model a free filament or a free solid body, respectively, further highlighting the simplicity of the formulation. The general form of the CG formulation in equation (2.9) for an arbitrary number of solid bodies each with an arbitrary number of attached filaments is simply given by

2.10 $$M_{F}\left[\begin{array}{c} F\\ T \end{array}\right]=K,$$

where $M_{F}$ is analogous to the matrix in equation (2.9), F and T contain the forces and torques, respectively, exerted on all spheres by the fluid. The vector K is analogous to the right-hand side in equation (2.9) and defines the constitutive relations for every 3D Kirchoff filament in the system. In appendix B, we provide the explicit form of equation (2.10) and include a practical example of the CG matrix in the case of two free filaments. It is worth noting that the CG formulation in equation (2.10) is general and does not depend on the specific choice of hydrodynamic coupling. For this reason, equation (2.10) can be used in conjunction with a variety of computational and analytic methods for solving the fluid mechanics around slender bodies. In this paper, we exploit the mathematical capabilities of the RPY non-local hydrodynamic approximation [41] for low Reynolds number fluids.

### 2.2. Non-local hydrodynamic coupling and dimensionality reduction

We approximate the non-local hydrodynamic forces and torques on each interacting sphere using the RPY tensor [41]. This relates linear velocities **v**_*i*_ and angular velocities ***ω***_*i*_ with forces and torques acting on each sphere via the non-local hydrodynamic mobility matrix $M_{H}$, giving

2.11 $$\left[\begin{array}{c} V\\ \Omega \end{array}\right]=M_{H}\left[\begin{array}{c} F\\ T \end{array}\right],$$

where V and Ω contain the velocities **v**_*i*_ and angular velocities ***ω***_*i*_ of all spheres, respectively. The mobility tensor encodes the translational and rotational movement of each sphere, as well as the non-local coupling between spheres, depending on the sphere radius *a*_*i*_, fluid viscosity *η* and mutual distance, see appendix C for details. We note that as spheres begin to approach each other, the RPY approximation begins to break down, and thus additional lubrication corrections may need to be included. Zuk *et al*. [41] discuss the RPY approximation and its use in macromolecular bead models. The formulation presented here allows for any hydrodynamic method to be used instead of the RPY approximation, so depending on the specific problem a different hydrodynamic method may be employed.

Exploiting the invertibility of the non-local hydrodynamic mobility tensor above, the generalized CG formulation (equation (2.10)) for the *non-local* elastohydrodynamic coupling simply reads

2.12 $$M_{F}M_{H}^{-1}\left[\begin{array}{c} V\\ \Omega \end{array}\right]=K.$$

However, the governing equation (2.12) lists, unnecessarily, all linear **v**_*i*_ and angular ***ω***_*i*_ velocities for each filament’s spheres as unknowns. Within the same body–filament structure, translation and rotations of each sphere are coupled, as they are part of the same assembly. Hence, linear velocities can be written in terms of the angular velocities to reduce the dimensionality of a given body–filament structure (figure 2), see appendix D for details. As a result, **v**_*i*_ and ***ω***_*i*_ are a function of the velocity of the centre of mass of the body ${\dot{x}}_{b}$, its angular velocity ***ω***_*b*_, and the angular velocity of each segment $\omega_{i}^{\mathrm{seg}}$, from the same body–filament structure. By rigidly attaching the first segment to the body (figure 2), we set ***ω***_1_ = ***ω***_*b*_. Altogether, this reduces the dimensionality of the system from 6(*N*_body_ + *Nn*) to 3 + 3*N* in equation (2.12). The dimensionality reduction is achieved via matrix Q,

2.13 $$\left[\begin{array}{c} V\\ \Omega \end{array}\right]=QW,$$

where W is the reduced velocity system for all spheres in terms of $x_{b},\omega_{b},\omega_{i}^{\mathrm{seg}}$, for *i* > 1 (see appendix D for details). Substituting equation (2.13) into (2.12) gives the full non-local CG elastohydrodynamic system in 3D,

2.14 $$M_{F}M_{H}^{-1}QW=K,$$

where W contains the reduced unknown velocities of all interacting units of the elastohydrodynamic system. Knowledge of W provides linear and angular velocities of each sphere, but without the filament shape information. The filament shape, position and orientation can be found by numerically integrating these relative to the laboratory fixed frame of reference, for example, using the rate of change of the directors base d**d**_*i*_/d*t* = ***ω*** × **d**_*i*_. The latter, however, introduces severe accuracy issues due to preserving orthogonality and unit length of the directors [37]. We circumvent these difficulties in the next section by exploiting the exponential mapping of quaternions to accurately track rotations of the basis vectors in 3D.

### 2.3. Tracking director rotations in three dimensions using exponential mapping of quaternions

Euler angles are commonly used to track 3D rotations of a vector basis as a function of three parameters. The challenge of any three-parameter parametrization is the existence of coordinate-singularities. With Euler angles, this corresponds to performing two successive rotations about the same axis, effectively losing a degree of freedom, known as gimbal lock [42]. Coordinate-singularities can be circumvented by continuously changing the laboratory frame of reference [31], though this method is inappropriate for tracking multiple interacting filaments at once as we require here. Quaternions are canonically used to avoid coordinate-singularities by introducing four-parameter rotations, and have a history of use in the literature [9,43–45]. Using a naive quaternion implementation can increase considerably the computational time, as we detail in the results section. Instead, we focus our study on the exponential mapping of quaternions to track rotations in 3D (referred here as exponential mapping). The exponential map effectively re-parametrizes quaternions into a three-parameter rotation scheme, thus re-introducing singularities. However, unlike Euler angles, the coordinate-singularities of the exponential map are trivial to avoid, requiring only a rescaling rather than redefining entirely the laboratory frame of reference [42]. This enables faster and more efficient simulations of multiple interacting filaments and solid bodies via the CG formulation introduced above.

Next, we will briefly outline quaternions and exponential mapping, and their coupling with CG formulation. Quaternions are an extension of the complex plane into a complex four-dimensional space. A quaternion is given by $q=q_{0}+q_{1}i+q_{2}j+q_{2}k={\left[q_{0}\;q_{1}\;q_{2}\;q_{3}\right]}^{⊤}$, where *i*^2^ = *j*^2^ = *k*^2^ = *ijk* = −1. Unit quaternions can be used to rotate a vector basis **v** in space via **v**′ = **qvq**^−1^. The axis of rotation is given by the imaginary part of **q** and the angle of rotation by the real part of **q**. Specifically,

2.15 $$q=\left[\begin{array}{c} \cos(\frac{\theta }{2})\\ \sin\left(\frac{\theta }{2}\right)\hat{n} \end{array}\right],$$

gives the quaternion corresponding to a rotation about the axis $\hat{n}$ by *θ*. The angular velocity of a set of basis vectors is related to the quaternion by

2.16 $$\dot{q}=\frac{1}{2}\left[\begin{array}{ccc} -q_{1} & -q_{2} & -q_{3}\\ q_{0} & q_{3} & -q_{2}\\ -q_{3} & q_{0} & q_{1}\\ q_{2} & -q_{1} & q_{0} \end{array}\right]\omega ,$$

providing a simple framework for numerical integration of quaternions, though this increases the dimensionality of the system. Generalizing above to account for the multiple angular velocities of the system W, the state shape-vector $X_{\mathrm{quat}}$ evolves in time according to

2.17 $${\dot{X}}_{\mathrm{quat}}=CW,$$

where the matrix C is given in appendix E and is solved together with governing equation (2.14).

The exponential mapping, on the other hand, re-parametrizes quaternions back to a three-parameter parametrization [37]. We define a pure vector known as the generator **r**, which is related to a quaternion via the exponential map, $q=\exp(r)={\left[\cos\left|r\right|\;\text{sinc}\left|r\right|r^{⊤}\right]}^{⊤}$, where sinc x=sin⁡x/x. The angular velocity of a set of basis vectors defined by generator **r** can be written conveniently in matrix form [46],

2.18 $$\omega =2D\dot{r},$$

where the matrix **D** is given by

2.19 $$\begin{array}{rl} D & =\{\left[\begin{array}{ccc} 1 & 0 & 0\\ 0 & 1 & 0\\ 0 & 0 & 1 \end{array}\right]+\frac{\sin^{2}|r|}{|r{|}^{2}}\left[\begin{array}{ccc} 0 & -r_{3} & r_{2}\\ r_{3} & 0 & -r_{1}\\ -r_{2} & r_{1} & 0 \end{array}\right]\\ & \;-\frac{\left|r|-\sin|r|\cos|r\right|}{|r{|}^{3}}\left[\begin{array}{ccc} r_{2}^{2}+r_{3}^{2} & -r_{1}r_{2} & -r_{1}r_{3}\\ -r_{1}r_{2} & r_{1}^{2}+r_{3}^{2} & -r_{2}r_{3}\\ -r_{1}r_{3} & -r_{2}r_{3} & r_{1}^{2}+r_{2}^{2} \end{array}\right]\}. \end{array}$$

The determinant $\left|D|={\text{sinc}}^{2}|r\right|$ goes to zero as |**r**| → *nπ*, for integer *n* ≥ 1. As such, a rescaling is necessary whenever the magnitude of a generator approaches *nπ* to avoid coordinate-singularity [37]. This can be achieved by rescaling

2.20 $$r\to r-\pi \frac{r}{\left|r\right|},$$

whenever |**r**| ≥ *π*/2. This rescaling does not change the rotation performed by the generator **r** but keeps it far away from any coordinate-singularity. Equation (2.18) can be generalized to account for the angular velocities of each sphere in the system W so that the state shape-vector $X_{\mathrm{gen}}$ can be evolved in time using

2.21 $$W=D{\dot{X}}_{\mathrm{gen}},$$

which can be substituted, directly, into the governing system of equations (2.14). This provides a convenient matrix form to evolve in time the non-local, multi-filament-–body elastohydrodynamic ODE system

2.22 $$M_{F}M_{H}^{-1}QD{\dot{X}}_{\mathrm{gen}}=K.$$

After prescribing an initial state vector and boundary conditions, the governing system of equations, equations (2.14) and (2.17) for quaternions, and equation (2.22) for exponential mapping, can be solved directly for the time evolution of the system’s state vector. We approximate curvatures in K using finite differences of the director basis (using equation (2.1)). We use Matlab solver *ode15s* to handle numerical integration, but note that any ODE solver can be used, highlighting the simplicity of the matrix representation. A Matlab implementation is provided here [47] to serve as a basis for rapid customization and further generalizations. We hope that the simplicity of the matrix system allows researchers to use a numerical platform of their choice for quick implementation. For results taken using several parameter values, we make use of a high-performance computing system to run multiple simulations simultaneously.

## 3. Exponential mapping of quaternions and comparison with partial differentional equation formulations

We proceed with a comparison between the computational efficiency of quaternions and exponential mapping within the CG framework. We also compare our simulations with previous methods with similar levels of elastohydrodynamic accuracy [9,22], which have been equally validated against experiments, though employing distinct fluid–structure interaction approaches. Finally, in the next section, we provide exemplars of the capability of the method to a series of complex elastohydrodynamic systems in 3D that are straightforward to implement using the CG formulation.

We non-dimensionalize the system using length-scale *L*, timescale *T* given either by the characteristic frequency *ω* of the system or the relaxation time of the fibre, and force density *E*_*b*_/*L*^3^. For simplicity, we set *E*_*b*_ = *E*_*t*_. Finally, we consider any filament attachment to the body as a rigid connection, i.e. the filaments are clamped at body, meaning that the body and first segment of filaments rotate with equal angular velocity, and may not bend or twist relative to each other. The dimensionless system of equations is thus multiplied by the dimensionless stiffness parameter $S^{4}=L^{4}(\eta \omega /E_{b})$, where *η* is the fluid viscosity. The stiffness parameter is closely related to parameters such as the elastohydrodynamic number $E_{h}=8\pi S^{4}$, and the sperm number ${\mathrm{Sp}}^{4}=((\xi_{⊥}/\eta )S^{4})$, where *ξ*_⊥_ denotes the perpendicular resistive force drag coefficient from RFT used elsewhere [12,31], and characterizes the ratio of viscous and elastic forces in the system. For most problems, the stiffness parameter generally lies between S=1 and S=20. As the stiffness parameter is lowered in elastohydrodynamic systems, especially below S=1, solving the system becomes more numerically stiff and will take more time to solve.

**Figure 3.**
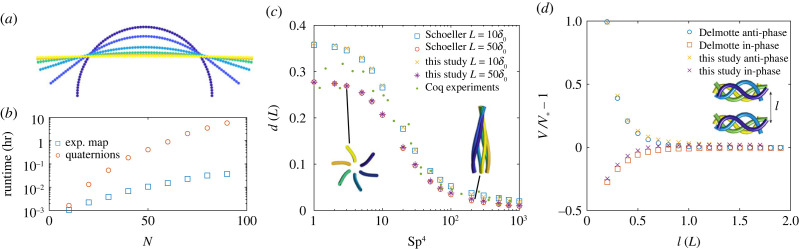
(*a*) Relaxation of filament with *N* = 40, *n* = 2 and S=3. Initial condition dark blue and shown at subsequent times *t* = 0.5, 1, 1.5, 2, 2.5, ending in a straight configuration. (*b*) Runtime in hours to solve for relaxation of single filament using quaternion and exponential map parametrization, for various number of segments *N*. Number of spheres per segment is *n* = 2 for all cases. (*c*) Distance that the end tip of helically actuated filament makes with the axis of rotation, averaged over time during steady state. Sperm number ${\mathrm{Sp}}^{4}=((4\pi )/(\log L/a+0.5))S^{4}$. Results agree well with simulations from Schoeller *et al.* [9] and experiments from Coq *et al.* [48]. (*d*) Swimming speed of two-swimmer system normalized by swimming speed of a single swimmer. Inset shows in-phase swimmers at several times over one period, with the initial distance between swimmers *l* labelled. Results show good agreement with data from Delmotte *et al.* [22].

We first compare the computational performance between quaternion and exponential mapping for the simple case of a filament free from external forces and torques, initially bent and undergoing relaxation dynamics, by recording the computational time taken to solve for the dynamics. Given no body exists, **x**_*b*_ = **x**_1_, **q**_*b*_ = **q**_1_ and **r**_*b*_ = **r**_1_. Initial quaternions are given by $q_{i}={\left[\cos(\theta_{i}/2)\;\sin\left(\theta_{i}/2\right){\hat{n}}^{⊤}\right]}^{⊤}$ and generators by $r_{i}=\theta_{i}/2\hat{n}$, where *θ*_*i*_ = (*i* − 1)*π*/(*N* − 1) and $\hat{n}=[0\;1\;0]$, for *i* = 1, …, *N*. This corresponds to a filament bent into a semi-circle, as shown in figure 3*a* for *N* = 40, *n* = 2. The initial shape unbends towards the unstressed straight configuration, with the centre of mass position conserved to within 2 × 10^−2^ (*L*). We set *n* = 2 and vary the number of segments *N*. Figure 3*b* shows the runtime in hours to solve from *t* = 0 to *t* = 20, for various values of *N* for both the quaternion and exponential mapping, with the stiffness parameter S=3. For *N* = 10 segments, runtimes are comparable at 3.8 s and 5.6 s for the exponential map and quaternions, respectively. As *N* increases, the exponential map results in significantly reduced runtimes. For example, at *N* = 30, runtimes are 13.7 s and 3 min for the exponential map and quaternions, respectively. This difference in runtime increases exponentially, and for *N* = 90 segments the exponential map parametrization runs over 150 times faster. This increase in runtime is true across all ranges of S. Hereafter, we proceed with our numerical study employing exponential mapping for more efficient computation.

In order to further test that deformations and displacements of the filament are being simulated accurately, we compare our results with experiments and simulations carried out by previous works. Schoeller *et al.* [9] simulate a single helically actuated filament, where one end of the filament is held a constant distance *δ*_0_ and angle *α*_0_ from an axis of rotation. The base of the filament is rotated at an angular velocity *ω* about the axis of rotation. We implement this using a filament with *N* = 20 and *n* = 1. Initial generators are all set to $r_{i}=\alpha_{0}/2{\left[0\;1\;0\right]}^{⊤}$. To enforce the motion of the base of the filament, we replace the force-free condition with the kinematic constraint ${\dot{x}}_{1}=-\delta_{0}{\left[\sin t\;\cos t\;0\right]}^{⊤}$, and the torque-free condition with the kinematic constraint ${\dot{r}}_{1}=(\alpha_{0}/2){\left[\cos t\;-\sin t\;0\right]}^{⊤}$. We solve the system between *t* = 0 and *t* = 10 000, ensuring the system reaches a steady state. Once a steady periodic state is achieved, we measure the distance that the tip of the filament makes with the axis of rotation, averaged over one period. Figure 3*c* shows the tip distance compared with results from Schoeller *et al*. [9] for *δ*_0_ = 0.1 (*L*) and *δ*_0_ = 0.02 (*L*) with *α*_0_ = 0.2618. Results agree excellently with both Schoeller *et al*. [9] and experimental data from Coq *et al*. [48]. It is worth noting that Schoeller *et al*. [9] has also been validated and contrasted with experiments independently. The agreement with other elastohydrodynamic methods in 3D, such as in Schoeller *et al*. [9], using the moment balance PDEs directly, together with Lagrange multipliers to enforce the inextensibility constraint, indicates equivalence between canonical PDE methods and the simpler CG method employed here in 3D, as also demonstrated in 2D by Moreau *et al*. [12].

We conduct one further test that places significance on the non-local hydrodynamics between spheres in the system. Following Delmotte *et al.* [22], we simulate the case of a single swimmer and then two identical swimmers coupled via non-local hydrodynamics. We prescribe a time-dependent intrinsic curvature to a filament made of *N* = 16 segments with *n* = 1 sphere per segment so that our hydrodynamic description matches the beads model employed in Delmotte *et al.* [22]. We use the curvature ***κ***^0^(*s*, *t*) = −*K*_0_sin(*ks* − *ωt*)**d**_1_(*s*, *t*), with *K*_0_ = 8.25, if *s* ≤ 0.5*L*, and *K*_0_ = 16.5(*L* − *s*)/*L*, if *s* > 0.5*L*, and a fixed stiffness parameter $S=22.6^{1/4}$. Numerical parameter values are chosen to match Delmotte *et al.* [22], which itself validated this swimming case with experiments [49] and simulations [50]. For the case of a single swimmer, the curvature wave causes planar beating that propels the filament forwards, characterized by a distance travelled per stroke of *V* = 0.0671 (*L*/*T*). This swimming speed compares well with *V* = 0.066 (*L*/*T*) from simulations in Delmotte *et al.* [22] and *V* = 0.07 (*L*/*T*) from experiments in Bilbao *et al*. [49].

We also measure the swimming speed of two active filaments swimming together, figure 3*d*, both with the same time-dependent curvature as the single swimmer above, but set to either in-phase or anti-phase (inset of figure 3*d* shows in-phase swimmers). The filaments are set an initial distance *l* apart and their initial configuration is taken from the steady state of the single swimmer case. The normalized swimming speed of the two-swimmer system is given in figure 3*d*, and shows a reduction in swimming speed for in-phase swimmers as they approach each other, and an increase in swimming speed for anti-phase swimmers as they approach each other. Figure 3*d* shows excellent agreement with Delmotte *et al.* [22] for in-phase and anti-phase swimmers. This is despite major differences in both modelling and computational formulations. In particular, Delmotte *et al.* [22] employs a novel gears model which solves the moment balance PDE with contact–contact forces between interlocked spheres making up the elastic filament which rotate relative to each other similarly to a gear, in such a way that the unknown contact forces play the role of Lagrange multipliers in the system. For anti-phase swimmers at small *l*, we used a modified version of the RPY tensors allowing for overlapping spheres given in Zuk *et al*. [41], as the beats of the two swimmers begin to overlap slightly. Using the modified version of the tensors ensures that the hydrodynamic mobility matrix remains positive-definite and non-singular if the separation between two spheres approaches zero, and does not affect runtime significantly. Additionally, as swimmers begin to touch, steric repulsive forces begin to play an important role, which may be added to the methodology, as we outline in the discussion.

## 4. Three-dimensional non-local elastohydrodynamic applications

### 4.1. Bi-flagellate *Chlamydomonas* elastohydrodynamic-swimming in three dimensions

*Chlamydomonas* is a widespread model organism whose flagella are structured similarly to those found in mammals, making it an important microorganism in experiments and modelling of flagella motion [51]. However, modelling *Chlamydomonas* is not an easy task due to the multiple elastic and solid interacting parts. It is composed of a solid body and two elastic tail-like appendages coupled by contact–contact and hydrodynamic interactions, while the whole system is free from external forces and torques during free-swimming motion. As such, *Chlamydomonas* modelling is often abstracted with minimal models [52–54]. There is a large amount of literature studying bi-flagellated swimmers using far from minimal models, with many prescribing the shape of the flagella in the body frame to solve for the swimming kinetics in the laboratory-fixed frame of reference, but not solving for the shape of the flagella [55–57]. There is also work where the full elastohydrodynamic system is solved; Fauci *et al.* [58,59] modelled *Chlamydomonas* using the immersed boundary method, and more recently Nourian & Shum [44] developed a numerical method to study bi-flagellated bacteria.

**Figure 4.**
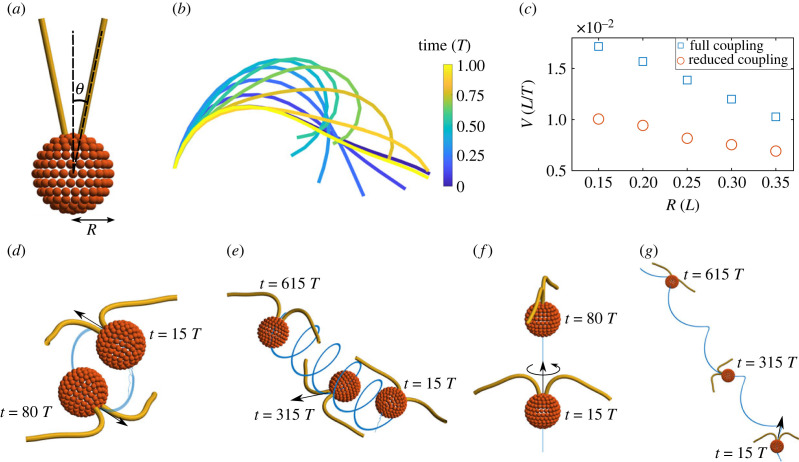
(*a*) Model *Chlamydomonas* set-up. Angle 2*θ* between two flagella attached to body of radius *R*. Here, 2*θ* = 23° and *R* = 0.35 (*L*). (*b*) Periodic bending wave given by time-dependent intrinsic curvature ***κ***^0^ = ±(4 + 4sin (2*πs* − *t*)) **d**_2_ shown over one period *T*, which induces straight swimming of *Chlamydomonas*. The **d**_2_ director basis along the entire length of both filaments points directly into the page, meaning the bending wave produced by this time-dependent curvature is perfectly in the plane of the page. (*c*) Swimming speed of *Chlamydomonas* dependent upon body radius and non-local hydrodynamic coupling. Reduced coupling indicates that terms in the RPY tensors coupling spheres in separate parts of the *Chlamydomonas* are set to zero (hydrodynamic coupling between flagellum–flagellum and flagellum–body are turned off). (*d*) Adding a constant out-of-plane curvature of magnitude $\kappa_{1}^{0}$ in the **d**_1_ direction causes out-of-plane swimming. Trajectory of *Chlamydomonas* when both flagella have equal out-of-plane intrinsic curvature $\kappa_{1}^{0}=0.5$. (*e*) Adding asymmetry between flagella turns the circular trajectory into a helical trajectory. Here, the flagella have intrinsic out-of-plane curvature $\kappa_{1}^{0}=0.5\pm 0.3$. (*f*) Trajectory of *Chlamydomonas* when both flagella have equal and opposite out-of-plane intrinsic curvature $\kappa_{1}^{0}=\pm 0.5$. (*g*) Asymmetric opposite sign out-of-plane curvatures $\kappa_{1}^{0}=\pm 0.5+0.3$ turn straight swimming with rolling into a helical trajectory, with rolling still present.

The coarse-grained *Chlamydomonas* swimmer is made of two active elastic filaments with an angle 2*θ* between them, attached to single spherical body of radius *R*, as depicted in figure 4*a*. The body is made out of *N*_body_ = 184 spheres and the filaments contain *N* = 15 segments with *n* = 1 sphere per segment. With S=3, we induce straight swimming by prescribing a time-dependent preferred curvature, $\kappa^{0}=\pm 4[1+\sin(2\pi s-t)]d_{2}(s)$ in each filament. We note that this curvature is the preferred curvature of the system that contributes to the bending moment, and the emergent waveform curvature depends on the total balance of moments on the flagella, as outlined in the Methods section. The constant term in the prescribed curvature induces a non-zero average curvature along the flagella (figure 4*b*), allowing the flagella to progressively ‘pull’ the spherical body over time, known as a puller swimmer. We vary the body radius (keeping *N*_body_ = 184) and record the swimming speed of the *Chlamydomonas* swimmer. Figure 4*c* shows the swimming speed of each *Chlamydomonas* as body size is increased with full non-local hydrodynamic coupling and reduced coupling (where hydrodynamic coupling between flagellum–flagellum and flagellum–body is turned off). Increasing the body size linearly decreases the swimming speed of *Chlamydomonas*, as expected from the linearity of Stokes flow. Reduced hydrodynamic coupling results in a slower swimming speed (up to 60% slower for the smallest body radius used here), highlighting the importance of non-local coupling for *Chlamydomonas* swimming (not possible with minimal models), and how this microorganism may exploit this effect to achieve higher swimming speeds. The anti-phase *Chlamydomonas* beating is thus critical to unlock higher propulsion, similarly to hydrodynamic coupled single-swimmers in figure 3*d*, that achieve higher speeds when moving in anti-phase near to each other. Interestingly, the difference in swimming speeds between the full and reduced hydrodynamic coupling decreases with the body size in figure 4*c*, indicating that Stokes law for the spherical body could provide a good approximation when the body is large (or the flagella are small). This is because the non-local hydrodynamic effect becomes increasingly small, when compared with the zeroth-order contribution from Stokes law, as the body size decreases.

Motivated by recent empirical studies observing three-dimensional beating of *Chlamydomonas* flagella [54,60], we show here that it is straightforward to implement 3D beating of *Chlamydomonas* using the CG framework. For the simplicity of this demonstration, we introduce a constant out-of-plane curvature $\kappa_{1}^{0}$ in the **d**_1_ direction of the two flagella, so that the time-dependent preferred curvature is $\kappa^{0}=\kappa_{1}^{0}d_{1}(s)\pm 4[1+\sin(2\pi s-t)]d_{2}(s)$. In the symmetric case, when $\kappa_{1}^{0}$ is the same for the two flagella, the out-of-plane beating causes the swimming path of *Chlamydomonas* to be no longer straight. Instead, the *Chlamydomonas* is trapped into perfect circular paths with radius *r*, linearly proportional to the mean out-of-plane curvature $\kappa_{1}^{0}$, shown in figure 4*d*. Interestingly, any incongruity in the mean intrinsic curvature between the two flagella is sufficient to allow *Chlamydomonas* to swim progressively via an array of helical paths in 3D, depending on differences in the orientation of their beating-planes, as observed experimentally [54,60].

By adding an asymmetry between the two flagella’s out-of-plane curvatures, the circular paths seen in figure 4*d* turn into helical paths shown in figure 4*e*. If instead the two out-of-plane curvatures are equal and opposite, the *Chlamydomonas* swims along a straight trajectory and rolls about its own axis, shown in figure 4*f*. Again, breaking the symmetry between the two flagella produces more complex swimming. In the case of opposite sign and asymmetric out-of-plane curvatures, the trajectory of *Chlamydomonas* becomes helical (distinct from the previous helical case; figure 4*g*), in further agreement with 3D observations [54,60].

### 4.2. Sperm–egg elastohydrodynamic scattering

The journey of sperm cells to the egg is one of the most important biological processes found in nature. The fertilization process involves the interaction of multiple bodies with varying size, active and passive components, and solid and elastic structures, providing endless opportunities in biological soft-matter and fluid–structure research [3]. Most of sperm swimming modelling focuses on hydrodynamic simulations using prescribed swimming beat patterns (kinematic constraints) to resolve swimming trajectories [61]. However, this bypasses the potential for elastohydrodynamic modulations in the system. In this example, we model for the first time the elastohydrodynamic scattering between a sperm and egg freely floating in the fluid. The CG formalism allows investigation of elastohydrodynamic swimming modulation and simple construction of elastic and passive solid bodies, that are fixed or free to move in the fluid. The latter is particularly challenging given that free force/torque balance governs the movement of the body as a whole. Here, we briefly test the hypothesis that spherical egg alone is able to hydrodynamically attract the sperm to the egg via non-local hydrodynamic interactions, and flagella bending re-orientation, as have been observed for bacteria swimming near spherical obstacles [62,63].

For simplicity, we model the sperm head with a spherical shape of radius 0.2*L* and the egg as a sphere of radius 1*L*. We note that eggs vary in size depending on the species [64]. The sperm flagella are made of *N* = 16 segments with *n* = 1 sphere per segment. Using an initial sperm–egg separation (measured from centre of sperm head to centre of egg) of 5*L*, we vary *θ*, the angle that the sperm cell makes with the *z*-axis at *t* = 0. Rather than prescribing a time-dependent intrinsic curvature, we prescribe an active internal moment density along the flagella. For this, we modify equation (2.3) accordingly, adding an extra term **m**_*a*_ which is the prescribed active moment density, **m**_*s*_ + **x**_*s*_ × **n** + ***τ*** + **m**_*a*_ = 0. When integrated, this modifies equation (2.7), giving an extra term on the right-hand side corresponding to the active moment density integrated from *s*_*j*_ to *L*,

4.1 $$\sum_{i=N_{\mathrm{body}}+(j-1)n+1}^{N_{\mathrm{body}}+Nn}((y_{i}-x_{j})\times F_{i}+T_{i})=-m_{j}-\int_{s_{j}}^{L}m_{a}\;ds.$$

**Figure 5.**
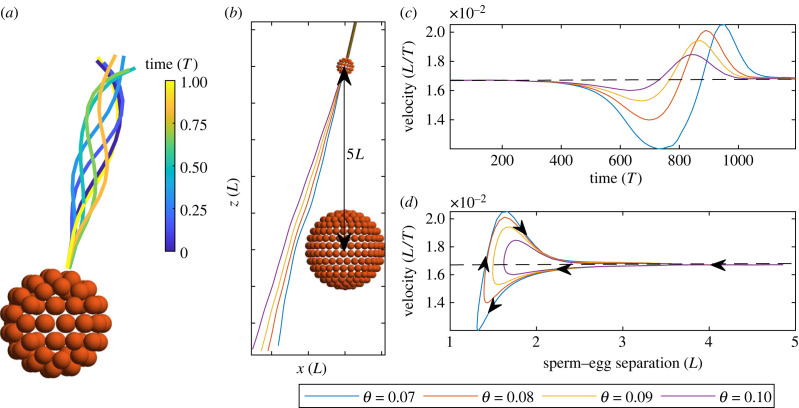
(*a*) Waveform of model sperm shown over one beat period T. (*b*) Trajectory of sperm past egg with different initial conditions for *θ*, the angle between the flagellum and $\hat{z}$. (*c*) Swimming speed dependent on time. Sperm slows down as it gets closer to the egg, and then begins to speed up as the sperm swims away from the egg. (*d*) Swimming speed dependent on sperm–egg separation, shown from *t* = 0 (*T*) to *t* = 1200 (*T*). Arrows indicate the flow of time. Depending on *θ*, the sperm reaches a minimum separation from the egg before swimming away and increasing in speed.

We set the active internal moment density **m**_*a*_ = 12*k* cos (*ks* − *t*)**d**_2_. This causes the waveform shown in figure 5*a*. Simulations are run with S=6. Figure 5*b* shows the initial condition for a single value of *θ*, and the subsequent trajectories of sperm cells towards the egg for four values of *θ*. The sperm cell that approaches closest to the egg shows the most noticeable curve in trajectory, as well as the largest change in speed as it swims past the egg (figure 5*c*).

Figure 5*d* shows the sperm swimming speed as a function of sperm–egg separation; initially separated by 5*L*, the sperms approach the egg and their swimming speed slows. This reduction in swimming speed is up to 28% for the sperm with the closest approach. Once the sperm reaches the closest separation from the egg, its swimming speed abruptly increases before gradually returning to its free-space swimming speed (i.e. the swimming speed in absence of solid bodies), as the sperm–egg separation increases. These simulations indicate that hydrodynamic effects alone are unable to assist sperm to find eggs with similar sizes as the sperm, even when swimming in close proximity. This may explain the need for other form of guidance mechanisms through evolution, such as chemotaxis in freshwater fish species [65]. Most interestingly, figure 5*d* seems to indicate the possibility of accelerating microorganisms by solid body scattering, though this depends on the proximity of the swimmer to the free body. The curves in figure 5*d* are skewed, and above the average speed for certain sperm–egg separations, so that the accumulated distance travelled is higher for those cases (see purple and yellow curves in figure 5*d*). We highlight that similar effects of swimmer–surface interactions have been discussed previously [61,66–69], and specifically speed-modulation of swimmers due to boundaries in [70]. In all, the exemplary results in figure 5 motivate further studies on the design and optimization of micro-environments to promote, or suppress, cell transport and progression using microfluidic systems.

### 4.3. Elastohydrodynamic cilia array and particle transport

**Figure 6.**
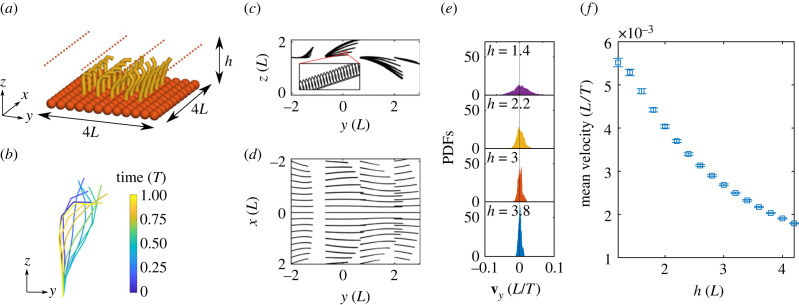
(*a*) Initial condition for 5 × 5 cilia array with wall made of spheres and tracer particles positioned above. Tracer particle radius *r* = 0.01 (*L*) (plotted larger for visual purposes). Cilia beat in the *y*–*z* plane and induce net fluid flow in the positive *y*-direction. The phase of cilia beating varies by 2*π* across the array, with cilia at the same *y* having identical phase. Tracer particles pushed by fluid flow created by cilia array. (*b*) Beating of a single cilium over one period with time progressing from blue to yellow. (*c*) Side-view of trajectory of tracer particles. Their direction of travel is from negative *y* to positive *y*. (*d*) Birds-eye view of trajectory of tracer particles. (*e*) Probability density functions (PDFs) of tracer particle velocities in the *y* direction, for several values of initial height *h*. (*f*) Particles that are initially higher up in *z* are further from cilia array and as a result have lower average velocity in the *y*-direction.

Arrays of cilia are found in many places in our body and are responsible for a wide array of biological processes, from driving fluid flow during embryonic growth to clearing mucus in the lungs [5]. Elastohydrodynamic simulations of cilia array have been developed for decades to investigate several multi-scale aspects of this system, including studies on synchronization phenomena and metachronal waves [71–75]. Here, we consider this canonical system using the CG formalism for the first time. The method outlined here is capable of simulating arrays of cilia and tracking fluid flow, which we demonstrate with a simple example. We arrange 25 cilia into a 5 × 5 grid arranged on top of a wall made of spheres, as shown in figure 6*a*. Each cilium is made of *N* = 7 segments with *n* = 1 sphere per segment and governed by a prescribed curvature ***κ*** = (1 + 5sin(1.5*πs* − *t* + *ϕ*)) **d**_1_, figure 6*b*. Each row (cilia with same location in *y*) in the cilia array has a different value for the phase *ϕ* such that a metachronal wave is travelling in the positive *y* direction, figure 6*a*. Specifically, *ϕ* = (*i* − 1)/5 × 2*π* where *i* corresponds to the row number of the cilium, for *i* = 1, …, 5. The beating of each individual cilium is shown in figure 6*b* over one period of oscillation. We note that one may use modified mobility tensors accounting for the hydrodynamics of a wall, which would speed up computation, but we choose to use spheres to model the wall for generality. In our example, the wall is fixed, but is easily made free, and can also be transformed into a non-planar ciliated surface (e.g. a curved wall) to investigate the effect of different wall geometries [73], something that is not immediately possible using modified hydrodynamic tensors.

The metachronal wave induces a net fluid flow above the cilia array, which transports tracer particles positioned above the array. The resultant trajectory of tracer particles with an initial height of 1.4 (*L*) can be seen in figure 6*c*,*d* from a side view and birds-eye view, respectively. The inset in figure 6*c* shows the oscillations of a single tracer particle over the beating period, and correlates with the ciliary beating driving them. Over long time, the tracer particles are successfully transported to the positive side of the *y* axis, in accordance with the biased waveform in figure 6*b*, with particles near the centre of the array moving faster than those at the sides due to the finite size of the array. Other boundary effects of the patch of cilia can be seen in figure 6*c*, in which particles tend to move upward (downward) for negative (positive) *y*. The probability density function (PDF) of particle velocity in the *y*-direction for different initial heights in figure 6*e* shows that the particle transport is not effective for this ciliary beating. The transport velocity is only weakly shifted to positive values due to the small component of the beating in the direction of transport, with the average velocity of transport decaying with particle height, shown in figure 6*f*. Distributions of accessible velocities by the tracer particles vary noticeably with particle height due to the fast spacial characteristic decay of the velocity field in low Reynolds number.

## 5. Discussion and conclusion

We offer a simple and intuitive method for simulating fully 3D interacting elastic filaments coupled via non-local multi-body microhydrodynamics (Matlab code provided [47]). By using asymptotic integration of momentum balance along each coarse-grained segment [12], we avoid challenging numerical treatment of high-order fluid–structure interaction PDEs and the numerically stiff Lagrange multipliers required to enforce the inextensibility constraint. Our method allows straightforward construction of complicated multi-filament–body systems involving moving, fixed and coupled-filament conditions, allowing easier treatment of cumbersome force-free and torque-free constraints. Our CG formulation features the construction of complex solid body geometries using spheres as building blocks, and the exponential mapping of quaternions to overcome coordinate-singularities in 3D with a trivial rescaling.

The CG framework recasts the 3D non-local elastohydrodynamic PDEs into an ODE system of equations, bypassing altogether the need of numerically meshing partial derivatives. This allows the use of generic numerical solvers. As such, no experience is needed in numerical methods, and only basic knowledge of linear algebra and matrix manipulation are required to use this framework. The full elastohydrodynamic system is simply condensed into block-operators,

5.1 $$M_{F}M_{H}^{-1}QD{\dot{X}}_{\mathrm{gen}}=K,$$

each encoding distinct model interactions of the shape field ${\dot{X}}_{\mathrm{gen}}$, namely: momentum balance $M_{F}$, hydrodynamic coupling $M_{H}^{-1}$, dimensional reduction Q, geometry of deformation and basis tracking D, and constitutive relations, internal activity and other boundary constraints K. Equation (5.1) is succinct and facilitates model explainability, generalizations and/or simplifications, as well as analytical and numerical analysis, better treatment of boundary conditions and easier implementation on different platforms. The formalism allows model customization without the need of algorithmic or numerical redesign. For example, the reduction in dimensionality operator may be removed if needed, the non-local hydrodynamic block may be replaced by other local or non-local drag theories, or by numerical solutions from a Navier–Stokes solver (e.g. immersed boundary methods). The exponential mapping of quaternions may be replaced by other coordinate parametrizations of choice, and different material properties of the filament may be equally invoked.

Adding non-trivial biologically inspired features of microswimmers is also straightforward with this formalism. For example, the elastic bending moment in equation (2.8) can be modified to allow for regions of different bending stiffness; the bending stiffness *E*_*b*_ may be a function of arclength or depend on the plane of bending. Regions of inactivity may be included by, for example, making the active moment density **m**_*a*_ in equation (4.1) zero for some range in arclength. If using corrections to the RPY tensors that allow for overlapping spheres, sphere radii can be readily changed along the length of the flagellum to account for varying filament radius. Segments could comprise spheres that are placed off the centreline, to incorporate unusual flagella geometries. Including additional forces and torque is also fairly straightforward. For example, they can be added to the right-hand side of the system of equations, giving

5.2 $$M_{F}M_{H}^{-1}QD{\dot{X}}_{\mathrm{gen}}=K-M_{F}\left[\begin{array}{c} F_{*}(X)\\ T_{*}(X) \end{array}\right],$$

where $F_{*}(X)$ and $T_{*}(X)$ contain additional forces and torques on spheres that are functions of the current state of the system. These may include repulsive steric forces that stop structures from overlapping, or attractive forces between spheres on different structures that bind filaments to other filaments or solid bodies. These forces can be switched on or off as a function of sphere separation or force magnitude, to incorporate the effect of new elastic bonds forming or breaking beyond a force critical value. Other types of forces arising from, for example, gravity, magnetism, electrostatic and stochastic interactions are straightforward to implement, offering further flexibility while employing this methodology.

The method has been validated and contrasted against previous experimental and computational studies showing excellent agreement with the literature (figure 3). We show that the exponential mapping parametrization provides a simpler implementation, and generally faster running times, than a direct quaternion implementation, but both parametrizations are robust while tracking multiple interacting filaments. There are several reasons why the direct quaternion implementation runs slower than the exponential mapping in our framework. First and foremost, the quaternion parametrization requires solving for angular velocities first, and then calculating for the rate of change of quaternions. In the exponential mapping implementation, we solve directly for the rate of change of the exponential map. A large contribution to the runtime of the quaternion implementation is due to the variable timestep solver in Matlab, which adjusts the timestep in order to achieve the desired tolerance. With the quaternion parametrization, equation (2.16) implicitly contains the constraint that the quaternion remains unitary, whereas the exponential map gives a unit quaternion by definition, allowing larger timesteps in the exponential map implementation with the same tolerances.

We showcase the method in three complex elastohydrodynamic examples: (i) bi-flagellated *Chlamydomonas* swimming in 3D (figure 4), (ii) sperm-egg elastohydrodynamic coupling (figure 5), and (iii) canonical particle transport by cilia array (figure 6). The elastohydrodynamic models and results presented for examples (i) and (ii) are novel. Indeed, the computational realization of these systems using the classical PDE formulation is still challenging today, specifically regarding the implementation of the boundary conditions and other filament constraints.

Our simulations revealed that the non-local hydrodynamic coupling increases the elastohydrodynamic propulsion of the *Chlamydomonas* swimming. Most importantly, the 3D elastohydrodynamic model is able to predict a bewildering array of complex 3D swimming trajectories that may arise via simple symmetry-breaking features of the flagella beat in 3D, from circular to helical trajectories (figure 4), in agreement with [54,60]. This offers numerous modelling opportunities motivated by recent 3D observations of this important model microorganism [54,60]. We also report novel sperm-scattering results in which sperm swimming can either be enhanced or reduced by the presence of a free body (figure 5). Finally, we observe canonical small-scale oscillations of tracer particles in synchrony with ciliary beating, and show that the non-local particle transport decays with height (figure 6), thus mimicking generic properties of ciliary systems, for the first time using the CG framework.

Numerous open questions exist in elasto-microhydrodynamics systems in which this method could be readily employed: self-organization of flagellar beat [76,77], elastohydrodynamic modulation of active bundles [78], non-trivial body geometries [79], physicochemical active guidance [65], ciliated microorganisms [80], cilia carpets [81], artificial swimmers [82,83], soft-robotics [84,85], locomotive robotics [86] and the role of torsional instabilities in filament systems [87], to name a few. Extending the framework to include inertial forces or the full Cosserat rod theory would widen the scope for the method’s use. As the size of the system becomes very large, methodologies which are optimized for large systems [9] will probably be more computationally efficient, but we note that significant runtime improvements could be achieved by tailoring the numerical scheme to specific applications, for example, using adaptive coarse-graining similar to the adaptive mesh used in [88], reducing hydrodynamics to RFT in single swimmer studies as in [27], in combination with using specific integration schemes [89] rather than built-in solvers used here, as well as using features such as Matlab MEX functionality to calculate the hydrodynamic mobility matrix. In all, we hope that the ease-of-implementation advantages of the method will facilitate the study of non-trivial fluid–structure interaction systems and assist researchers to fast-track implementation and accelerate the modelling cycle, serving communities within, and away from, mathematical and physical sciences that are equally interested in simulating these systems.

## Data Availability

The Matlab codes used to run the simulations presented in this paper are available on github via the link: https://github.com/polymaths-lab/3d-coarse-graining-elastohydrodynamics or on Zenodo at: https://zenodo.org/record/7940817 [47].

## Appendix A. Matrix cross product

The cross product **a** × **b** in matrix form is given by

A 1 $$a\times b={\left[a\right]}_{\times }b=\left[\begin{array}{ccc} 0 & -a_{3} & a_{2}\\ a_{3} & 0 & -a_{1}\\ -a_{2} & a_{1} & 0 \end{array}\right]\left[\begin{array}{c} b_{1}\\ b_{2}\\ b_{3} \end{array}\right].$$

## Appendix B. Force and torque balance of two free filaments

For a single filament with no body, the block of zeros in the large matrix in equation (2.9) reduces to zero size, and the number of spheres in the system is given by *M* = *Nn*. If **M**_*F*_ encodes the force and torque balance of a single filament, e.g.

B 1 $$M_{F}\left[\begin{array}{c} F\\ T \end{array}\right]=K,$$

then the force and torque balance of two free filaments is given by

B 2 $$\left[\begin{array}{cc} {\left(M_{F}\right)}_{1} & 0\\ 0 & {\left(M_{F}\right)}_{2} \end{array}\right]\left[\begin{array}{c} F_{1}\\ T_{1}\\ F_{2}\\ T_{2} \end{array}\right]=\left[\begin{array}{c} K_{1}\\ K_{2} \end{array}\right],$$

where (**M**_*F*_)_*i*_, $F_{i}$, $T_{i}$ and $K_{i}$ have identical structure to the single filament case and *i* = 1, 2 denotes the filament number. When writing in general form

B 3 $$M_{F}\left[\begin{array}{c} F\\ T \end{array}\right]=K,$$

rows and columns in equation (B 2) are rearranged accordingly. Adding additional structures results in adding additional matrices along the diagonal in equation (B 2). For example, adding a single free sphere to the system would add six additional rows to $M_{F}$, corresponding to one identity matrix multiplying the force experienced by the sphere, and one identity matrix multiplying the torque experienced by the sphere. Six extra zeros would be added to the right-hand side corresponding to the force- and torque-free conditions.

## Appendix C. Hydrodynamic mobility matrix

Using the RPY approximation gives the hydrodynamic mobility matrix

C 1 $$M_{H}=\left[\begin{array}{cc} \mu^{tt} & \mu^{tr}\\ \mu^{rt} & \mu^{rr} \end{array}\right],$$

where *t* and *r* refer to translational and rotational components, respectively. The submatrices have general form

C 2 $$\mu =\left[\begin{array}{cccc} \mu_{11} & \mu_{12} & \ldots & \mu_{1M}\\ \mu_{21} & \mu_{22} & \ldots & \mu_{2M}\\ \vdots & \vdots & \ddots & \vdots \\ \mu_{M1} & \mu_{M2} & \ldots & \mu_{MM} \end{array}\right].$$

Components of ***μ***^*tt*^, ***μ***^*tr*^, ***μ***^*rt*^ and ***μ***^*rr*^ are given by [41]

C 3 $$\mu_{ij}^{tt}=\left\{ \begin{array}{cc} \frac{1}{6\pi \eta a_{i}}I\; & i=j,\;\\ \frac{1}{8\pi \eta r_{ij}}(\left(1+\frac{a_{i}^{2}+a_{\;j}^{2}}{3r_{ij}^{2}}\right)I\;\\ \;+\left(1-\frac{a_{i}^{2}+a_{\;j}^{2}}{r_{ij}^{2}}\right){\hat{r}}_{ij}{\hat{r}}_{ij})\; & i\neq j,\; \end{array}\right.$$

C 4 $$\mu_{ij}^{rr}=\left\{ \begin{array}{cc} \frac{1}{8\pi \eta a_{i}^{3}}I\; & i=j,\;\\ \frac{1}{16\pi \eta r_{ij}^{3}}(3{\hat{r}}_{ij}{\hat{r}}_{ij}-I)\; & i\neq j,\; \end{array}\right.$$

C 5 $$\mu_{ij}^{tr}=\mu_{ij}^{rt}=\left\{ \begin{array}{cc} 0\; & i=j,\;\\ \frac{1}{8\pi \eta r_{ij}^{2}}\varepsilon \cdot {\hat{r}}_{ij}\; & i\neq j,\; \end{array}\right.$$

where *a*_*i*_ is the radius of sphere *i*, *η* is the fluid viscosity, **r**_*ij*_ is the separation of sphere *i* and *j*, $r_{ij}=\|r_{ij}\|$, ${\hat{r}}_{ij}=r_{ij}/r_{ij}$ and ε is the Levi–Civita symbol. When *i* = *j*, $\mu_{ij}^{tt}$ and $\mu_{ij}^{rr}$ reduce to standard force and torque relations for a sphere in the Stokes regime, and there is no translation–rotation component. For *i* ≠ *j*, the tensors dictate how sphere *j* affects the movement of sphere *i*. For example, $\mu_{ij}^{tt}$ couples the force on sphere *j* with the velocity of sphere *i*; $\mu_{ij}^{tr}$ couples the torque on sphere *j* with the velocity of sphere *i* and $\mu_{ij}^{rr}$ couples the torque on sphere *j* with the angular velocity of sphere *i*. The tensors used here allow for spheres of different size, allowing different structures to be constructed out of differently sized spheres, depending upon the precision required.

## Appendix D. Reducing dimensionality of system

Consider a single filament attached to a solid body. Spheres are rigidly attached within each segment, and the velocity at **x**_*i*_ is given by the velocity at **x**_*i*−1_ plus a contribution from the angular velocity of segment *i* − 1. Additionally, the velocity of spheres in the body are given by the velocity ${\dot{x}}_{b}$ plus a contribution from the angular velocity of the body ***ω***_*b*_. As such, the velocity of a sphere in the system is given by

D 1 $$v_{i}=\left\{ \begin{array}{cc} {\dot{x}}_{b}-\Delta_{i}\times \omega_{b}\; & i\leq N_{\mathrm{body}},\;\\ {\dot{x}}_{b}-(x_{1}-x_{b})\times \omega_{b}-\Delta s\sum_{\;j=1}^{\lceil k/n\rceil -1}\; & \;\\ \;d_{3}^{j}\times \omega_{j}^{\mathrm{seg}}-\frac{\Delta s}{2n}(2l-1)d_{3}^{\left\lceil k/n\right\rceil }\times \omega_{\left\lceil k/n\right\rceil }^{\mathrm{seg}}\; & i>N_{\mathrm{body}},\; \end{array}\right.$$

where Δ*s* is the length of each segment, *k* = *i* − *N*_body_, ⌈k/n⌉ rounds *k*/*n* up to the nearest integer, l=k−n(⌈k/n⌉−1) and $\omega_{i}^{\mathrm{seg}}$ is the angular velocity of the *i*th segment in the filament. The quantity ⌈k/n⌉ gives the segment number that sphere *i* is attached to and *l* gives the sphere number on that segment (e.g. *l* ≤ *n*). The angular velocity of a sphere in the system is given by

D 2 $$\omega_{i}=\left\{ \begin{array}{cc} \omega_{b}\; & i\leq N_{\mathrm{body}},\;\\ \omega_{\left\lceil k/n\right\rceil }^{\mathrm{seg}}\; & i>N_{\mathrm{body}}.\; \end{array}\right.$$

Equations (D 1) and (D 2) allow all **v**_*i*_ and ***ω***_*i*_ in the system to be written in terms of velocity of the centre of mass of the body ${\dot{x}}_{b}$, its angular velocity ***ω***_*b*_ and the angular velocity of each filament segment $\omega_{\;i}^{\mathrm{seg}}$. By additionally rigidly attaching the first segment of the filament to the body, the dimensionality of the system is reduced from 6(*N*_body_ + *Nn*) to 3 + 3*N* velocities. Writing equations (D 1) and (D 2) in matrix form gives equation (2.13) in the main text.

## Appendix E. Implementing quaternion parametrization

Consider again the single filament attached to a body. Assign a quaternion **q**_*b*_ to the body frame vectors and **q**_*i*_ to filament segment frame vectors. The state vector for the system is

E 1 $$X_{\mathrm{quat}}={\left[\begin{array}{ccccc} x_{b}^{⊤} & q_{b}^{⊤} & q_{1}^{⊤} & \ldots & q_{N}^{⊤} \end{array}\right]}^{⊤}.$$

The rate of change of the state vector is given by

E 2

where **P**(**q**) is given by (see equation (2.16))

E 3 $$P(q)=\frac{1}{2}\left[\begin{array}{ccc} -q_{1} & -q_{2} & -q_{3}\\ q_{0} & q_{3} & -q_{2}\\ -q_{3} & q_{0} & q_{1}\\ q_{2} & -q_{1} & q_{0} \end{array}\right].$$

By rigidly attaching the first segment of the filament to the body, $\omega_{1}^{\mathrm{seg}}=\omega_{b}$, the dimension of the vector on the right-hand side of equation (E 2) can be reduced by 3, in addition to removing one column from the matrix pre-multiplying the vector. Generalizing to arbitrary systems gives the dynamics of the state vector $X_{\mathrm{quat}}$ in terms of the reduced velocity system W via the matrix C,

E 4 $${\dot{X}}_{\mathrm{quat}}=CW.$$
