# Correction: ‘The three-dimensional coarse-graining formulation of interacting elastohydrodynamic filaments and multi-body microhydrodynamics’ (2023), by Fuchter and Bloomfield-Gadêlha

**DOI:** 10.1098/rsif.2024.0800

**Published:** 2024-12-04

**Authors:** Paul Fuchter, Hermes Bloomfield-Gadêlha

**Affiliations:** ^1^ School of Engineering Mathematics and Technology, University of Bristol, Bristol, UK

**Keywords:** elastohydrodynamics of filaments, microhydrodynamics, fluid–structure interaction, elastic filaments in three dimensions, cilia and flagella, microorganisms and microswimmers

J. R. Soc. Interface 20:2020230021 (Published online 31 May 2023). (https://doi.org/10.1098/rsif.2023.0021)

The paper omitted the grant number details in the previous funding information. The funding information should read:

This work was supported by an Engineering & Physical Sciences Research Council DTP [grant number EP/T517872/1].

